# Multiprofessional screening protocol for dysphagia in patients with HIV infection: elaboration and content validity

**DOI:** 10.1590/2317-1782/20212021012

**Published:** 2021-10-22

**Authors:** Luise Alexandre Rocha Soutinho, Daniel Aragão Machado, Charles Henrique Dias Marques

**Affiliations:** 1 Universidade Federal do Rio de Janeiro – UFRJ - Rio de Janeiro (RJ), Brasil.; 2 Universidade Federal do Estado do Rio de Janeiro – UNIRIO - Rio de Janeiro (RJ), Brasil.

**Keywords:** Deglutition Disorders, Protocols, Mass Screening, Acquired Immunodeficiency Syndrome, HIV Infections

## Abstract

**Purpose:**

To elaborate and validate a multiprofessional protocol to identify the risk of dysphagia in people with HIV at the time of hospitalization.

**Methods:**

After bibliographic review, the dysphagia screening protocol created was submitted to the analysis of HIV/ Aids expert judges and target audience in the application of this instrument to perform content validity. These evaluators could suggest changes to the protocol, judging clarity, pertinence, and comprehensiveness. The CVI 0.78 was used to confirm the validity of the results.

**Results:**

The protocol was created including aspects related to oral and pharyngeal swallowing, and the final score was calculated based on the risks for clinical complications. The instrument presented CVI above 0.78 for all items in the two validation phases, as well as total CVI of 0.92.

**Conclusion:**

Based on the obtained data, it was possible to create and validate the screening protocol from the point of view of appearance and content, once it presented total CVI above the minimum value stipulated in the validation of the expert judges and the target public, obtaining an adequate result for the protocol. Therefore, we can consider the resolution instrument, with the capacity to fulfill what was proposed.

## INTRODUCTION

The first cases of the Acquired Immunodeficiency Syndrome (AIDS) were in the United States, Haiti, and Central Africa in the late 1970s as result of an infection by the human immunodeficiency virus, HIV, which affects the cells of the immune system^(1)^.

Since the discovery of the first cases of this infection until the end of the 1990s, the World Health Organization (WHO) estimates that around 29.4 million people have been infected with HIV^(2)^.

Brazil identified the first cases only in the 1980s, and only in 1983, the first AIDS control program in the country was structured^(3)^. According to the epidemiological bulletin, more than 650 thousand cases of AIDS in the first 30 years were reported in the SINAN (Information System for Notifiable Diseases^(4)^.

Virus infection results in a wide range of clinical manifestations, ranging from asymptomatic individuals to symptoms such as weakness, fever, weight loss, and prolonged diarrhea, candidiasis, neurotoxoplasmosis, among others^(5)^. During the HIV infection, the virus enters the central nervous system (CNS) and can cause cognitive function disorders, leading to deficits in processes such as attention and memory, and dementia and motor symptoms^(6)^.

Manifestations in the oropharyngeal region, with candidiasis as the most common cause, may present symptoms such as oral pain, bitter or sour taste in the mouth, or even esophagitis with odynophagia (pain when swallowing)^(5)^. Because of these symptoms, we can infer that this individual can present from speech articulation disorders due to injuries to swallowing difficulties (dysphagia).

Swallowing is a neuromuscular activity that transports food from the oral cavity to the stomach, involving the coordination of structures that participate in both swallowing and breathing, not allowing substances to enter the airways^(7)^. Dysphagia is a disorder during this process, defined as a symptom, in which the change in swallowing is due to some underlying disease.

Although the swallowing phases are didactically divided into oral, pharyngeal, and esophageal phases, swallowing is a continuous process. Therefore, any commitment in just one of these phases can influence the others, leading to losses in the entire process.

As HIV infection progresses in infected individuals, there may be a gradual reduction in lean body mass, which is associated with a reduction in the capacity of the musculoskeletal system to generate strength^(8)^. Consequently, the functional performance of structures related to swallowing is affected, as swallowing is a function performed by effort. In addition to this hypotony, the oral microbiota of HIV-positive patients is different (they show an increase in yeasts of various species) from the oral microbiota of immunocompetent individuals^(1)^. When associated with the patient's low immunity, it leads to the appearance of oral manifestations, which are closely related to significant impairments in the oral phase of swallowing^(9)^.

In a literature review, we found a study that reports dysphagia symptoms in 47% of AIDS patients^(10)^. These damages related to the oral phase of swallowing influence not only the ejection of the bolus but also the sequentially of the process. When there is esophageal involvement, this incidence rises to 59 to 79% of patients^(4)^.

Multiprofessional care is essential for individuals with dysphagia, considering that its consequences involve clinical aspects, in addition to possible impacts on the quality of life and on the social aspects of eating, leading to the individual's retraction and isolation.

In the swallowing assessment, we need to know how some diseases, manifested in the individual with HIV, influence the swallowing function and its phases. The elaboration of protocols that guide the action of the multidisciplinary team aims to guarantee the quality of the service offered, defining the actions to be taken.

In a previous study, there was a reduction in the incidence of aspiration pneumonia in hospitalized patients, based on the application of a formal dysphagia assessment protocol^(11)^. Early identification of the difficulty and early referral to the Speech-Language Pathologist aim to avoid episodes of bronchoaspiration and, consequently, reduce the length of hospital stay and the risks of infection, promoting a reduction in the costs of the health system and providing a better quality of life for these patients.

This study aimed to create a multidisciplinary protocol to identify the risk of dysphagia in people living with HIV at the time of hospitalization. Also, it aimed to validate the protocol, with experts in the care of people living with HIV and with the multidisciplinary team, with ways to validate the agreement of the target audience.

## METHODS

This is a descriptive study, with a quantitative approach, which aims to create a product and its validation to make it reliable and valid for its intended purpose.

The instrument created in this study consists of a questionnaire validated and applied by several health professionals, specifically in patients diagnosed with HIV, aged at least 18 years old at the time of their hospitalization.

The study was divided into two stages: the creation of the questionnaire and its content-based validation.

The first stage included a literature review on the use of dysphagia screening protocols and questionnaire validation methods. This review allowed us to analyze the protocols already validated to adapt their questions and create a new specific protocol for people living with HIV.

We performed advanced searches in the BVS and PUBMED databases. As for temporality, we included studies published between 1984 and 2015, using the descriptors “acquired immunodeficiency syndrome”, “deglutition disorders”, “protocols”, “triage” and “mass screening”, as well as “face validity”, their combinations and their respective translations into Portuguese. Reviews and reference lists of all articles considered relevant were also consulted to include new articles. The search was performed using words found in titles, abstracts, and the body of the text.

The authors of the work elaborated the protocol. They are researchers who have experience in dysphagia and experience with patients with HIV.

At the end of this first stage, the protocol entitled: “Multiprofessional Dysphagia Screening Protocol in HIV Patients” (Appendix 1) was developed, which included aspects such as clinical risk criteria for oropharyngeal dysphagia and alterations in the oral and pharyngeal phase of swallowing.

In the second stage of the research, we used Face/Content Validation as it is a common method in the health area^(12)^. It is important that this process is carried out by professionals from different areas and that they are experts in the theme of the study, valuing the different suggestions and opinions on the topic^(13)^.

For this phase of content validation, the following guidelines were followed: choice of expert judges on the theme and their assessment of the individual items; the questionnaire as a whole and choice; and assessment of the target audience (professionals who will apply the questionnaire in clinical practice). All ratings were made using the Content Validity Index.

There is no consensus in the literature as to the ideal number of judges for the validation process, and the characteristics of the instrument should be taken into account^(14)^. Therefore, for this study, we used seven judges from different areas of health, who met the following inclusion criteria: minimum master's course in their areas and at least three years of clinical experience in caring for patients with HIV.

Each of these professionals invited to participate in the study received: an invitation letter with the objectives of the work, a screening protocol (Appendix 1), and a form for validation of appearance and content (Appendix 2). The protocol evaluation could be carried out in each evaluator's preferred location, with a period of fourteen days for returning the completed instrument.

The chosen judges were instructed to evaluate each of the 16 items of the questionnaire created for the study following these criteria: clarity of the written language used, the relevance of the subject in question, and coverage of aspects related to the topic addressed. For all items evaluated, they could include suggestions. Data collection was carried out in the last semester of 2017.

After evaluation and possible modification of the protocol by the experts, the material was judged by the target audience, that is, an evaluation regarding appearance, language, and applicability by professionals who will use this tool in individuals with HIV. It is an important moment for the study because we could verify the understanding of the population that will apply the protocol and analyze their suggestions. The appearance analysis stage aims to ascertain whether the material is understandable to the target population.

We recommended choosing 30 to 40 people from the target population for evaluation in this stage of the study^(15)^. Among these professionals, different components of the health team were selected, aiming to ensure the multidisciplinary character of the developed protocol.

The sample of the target audience was given by convenience and consisted of 36 health professionals working in a university hospital, following some inclusion criteria: being 18 years old or older; having time available to participate voluntarily in the evaluation of the screening protocol, and having work or student relationship with the hospital under study.

Again, we gave the following documents to everyone who agreed to participate in the research: a screening protocol (Appendix 1) and a form for validation of appearance and content (Appendix 2). We also requested to sign the Informed Consent Form - ICF.

The audience approached was instructed to evaluate each of the 16 items of the multiprofessional dysphagia screening protocol with the same criteria of clarity of written language, relevance, and scope of the subject in question. The evaluation of the protocol was carried out in a place chosen by the participants, with a period of one day for returning the completed instrument.

As well as the expert judges, we guided the target audience on how to fill out the questionnaire at different times, but it was done by the same individual, seeking to maintain equitable language and instruction, aiming to reduce possible distortions.

The Content Validity Index – CVI is designed to assess the percentage of judges who agree on a given item. It is considered valid if, after analyzing the judges' answers, it obtains an approval rate above 78% (0.78)^(16)^. To calculate the CVI for each item of the questionnaire (CVI - I), the total number of judges who assigned a score of 3 or 4 on a four-point ordinal scale from “irrelevant” to “extremely relevant” divided by the total of judges who participated in the evaluation^(16)^.

To assess the instrument (CVI – T), we used the average of the total number of items considered relevant by the judges, by the total number of items in the questionnaire. It was accepted for CVI approval – T above 90% (0.90)^(16)^.

Assessments below these values, both for each item and the total value of the test, were reviewed and sent back to the same judges, aiming for all to reach the minimum reliability value.

This stage is part of a broad instrument validation process. Thus, this phase covers the creation and acceptance of the questions used in the questionnaire by people who are experts on the theme and by health professionals who will apply the protocol in clinical practice. Only after this stage, we could use the instrument with individuals with HIV.

This project was submitted and approved by the Research Ethics Committee of the Federal University of the State of Rio de Janeiro - UNIRIO on August 30, 2017, under opinion number 2,247,883.

The ICF described all ethical aspects relevant to the research and participation in the study, which was signed after acceptance by the participants. Everyone was instructed about the privacy of the research data, guaranteeing that the information obtained could not be used for purposes other than those provided for in the project, and the possibility of refusing or interrupting their participation at any time, without any type of injury or penalty.

## RESULTS

The study researchers developed the multi professional protocol for screening for dysphagia in HIV patients based on the content of articles in the literature^(17-27)^ the authors' experience in formulating the division of items and scoring them.

We surveyed 21 articles, 3 in Portuguese and 18 in English. Sixteen of them had validated dysphagia assessment questionnaires, being only 11 with questions relevant to the study and 5 referring to validation methods. Based on these articles, we created the screening protocol considering aspects present in the dynamics of swallowing.

It was extremely important to transform the language of information located in scientific and specific literature into a language accessible to the target audience^(13)^. Therefore, all technical terms in the field of Speech-Language Pathology were replaced by synonyms that reached the meaning of the information.

The texts were written using a simple and easy-to-read font style, Arial font size 10 for information and 12 for the title. Graphic elements were also used to communicate information visually, in such a way as to show a step-by-step screening capable of locating the main risk factors for dysphagia and referral to the trained professional, in addition to arrows highlighting key information during the application of the protocol.

The screening protocol was divided into three parts:

1. Patient profile: showing data such as identification of the individual, data from previous exams, time since diagnosis, use of antiretroviral medication, weight, complaints, past pathological history, and identification of the professional who is applying the questionnaire;
2. Clinical risk criteria for dysphagia: indicating the presence of tracheostomy, need for oxygen support, difficulty in maintaining a level of alertness or adequate posture for eating, presence of lesions in the oral cavity, or complaints that represent a high risk for oropharyngeal dysphagia;
3. Signs and symptoms: including 16 questions created for this study that may represent some difficulty in the oral and pharyngeal phase of swallowing, leading this individual to a greater risk of having dysphagia.

It is important to emphasize that the completion of the protocol was interrupted when any item of clinical criteria was checked, as it places the individual at high risk for dysphagia, requiring early speech therapy assessment.

The final form of the protocol was titled: “Multiprofessional Protocol for the Screening of Dysphagia in HIV Patients” and its first version was sent to the expert judges. For this analysis, we chose professionals from the areas of Speech-Language Therapy, Nursing, Nutrition, and Medicine who have agreed to participate in the research.

Table 1 shows the profile of the 7 selected expert judges who validated the study material. According to the table, 6 of the judges are female (85.7%) and 1 male (14.3%). Regarding the age group, we observed that there is a greater concentration of experts in the range of 30 to 40 years old (57.1%), with a mean of 44.1 years old and a standard deviation (SD) of 11.67.

**Table 1:** Profile of expert judges who assess the screening protocol

**Variables**	**N**	**(%)**
**Gender**		
Female	6	-85.7
Male	1	-14.3
**Age**		
30-40 years old	4	-57.1
41-50 years old	1	-14.3
51 years old or more	2	-28.6
**Graduation**		
Speech-Language Therapy	3	-42.8
Nursing	2	-28.6
Medicine	1	-14.3
Nutrition	1	-14.3
**Time of graduation**		
Up to 15 years	3	-42.8
16-30 years	2	-28.6
31 years or more	2	-28.6
Titration		
Master's degree	4	-57.1
Doctorate	3	-42.9
**Work area**		
Teaching	--	--
Assistance	1	-14.3
Teaching and Assistance	6	-85.7
**Published articles**		
Yes	5	-71.4
No	2	-28.6

We followed an ordinal scale for each question, consisting of: “not relevant” (NR), “not very relevant” (NVR), “very relevant” (VR), and “highly relevant” (HR), which should be marked according to the criteria established above.

In the first part of the validation, all judges returned the completed questionnaires within the deadline, which were analyzed quantitatively, observing the proportions of the agreement described in Table 2.

**Table 2:** Dysphagia screening protocol content validation indexes - Judges - 1^st^ phase

**Instrument items of evaluation**	**Number of judges in agreement (n = 7)**	**CVI – I**
1 – Do you have dental elements?	6	0.86
2 – Do you use a dental prosthesis?	6	0.86
3 – Do you have any difficulty making movements with your face?	6	0.86
4 – Is it difficult to keep the food/liquid in the mouth? Does it escape or fall through the lips?	7	1.00
5 – Do you take longer to feed than before?	6	0.86
6 – Do you take a long time or find it difficult to eat hard foods?	5	0.71*
7 – After you swallow, are there remains of food in your mouth?	6	0.86
8 - Do you feel that you have a lot of saliva in your mouth or drool frequently?	7	1.00
9 – Do you feel pain when swallowing saliva, food, or liquids?	7	1.00
10 – Do you feel the food/liquid stuck in your throat?	7	1.00
11 – Do you need to swallow several times to feel that the food left your throat?	7	1.00
12 – Do you need to drink liquids to help the food go down?	6	0.86
13 – Do you feel that your voice changes during or after the meal?	6	0.86
14 – Do you cough or clear your throat during a meal?	6	0.86
15 – Do you feel suffocated or have difficulty breathing when eating?	7	1.00
16 – Does the food or drink go to the “wrong place” when you swallow it?	5	0.71*
	**CVI – T**	0.89

* Items that obtained CVI - I less than 0.78

After analysis and pertinent modifications, the 2^nd^ version of the protocol (Appendix 1) was sent to the expert judges so that they could judge the material, now modified, following the validation of appearance and content.

Table 3 shows the quantitative analysis of the agreement between the judges and the qualitative analysis of the suggestions, now with the modified protocol.

**Table 3:** Dysphagia screening protocol content validation indexes - Judges - 2^nd^ phase

**Instrument items of evaluation**	**Number of judges in agreement (n = 7)**	**CVI – I**
1 – Do you have dental elements?	6	0.86
2 – Do you use a dental prosthesis?	6	0.86
3 – Do you have any difficulty making movements with your face (Facial Paralysis)?	6	0.86
4 – Is it difficult to keep the food/liquid in the mouth? Does it escape or fall through the lips?	7	1.00
5 – Do you eat faster or slower than before the disease?	6	0.86
6 – Do you take a long time or find it difficult to eat solid foods after the illness?	6	0.86
7 – After you swallow, are there remains of food in your mouth?	7	1.00
8 - Do you feel that you have a lot of saliva in your mouth or drool frequently when you are awake?	6	0.86
9 – Do you feel pain when swallowing saliva, food, or liquids?	7	1.00
10 – Do you feel the food/liquid stuck in your throat?	7	1.00
11 – Do you need to swallow several times to help the food go down your throat?	7	1.00
12 – Did you start drinking liquids to help the food go down after the illness?	6	0.86
13 – Do you feel that your voice changes during or after the meal?	6	0.86
14 – Do you cough or clear your throat during a meal?	6	0.86
15 – Do you feel suffocated or have difficulty breathing when eating?	7	1.00
16 – Do you choke often?	7	1.00
	**CVI – T**	0.92

After this second specific phase of validation by the judges, the quantitative analysis showed CVI – I above 0.78 for all modified items of the screening protocol. Thus, the protocol can be considered partially validated, since it obtained a CVI - T of 0.92, being able to start the third phase of validation, which consists of evaluating the target audience.

The target audience for the evaluation of the protocol was composed of professionals from the area of Medicine and Nursing, and technicians, and university students from the courses, who worked in the first care of patients with the research profile, during their hospital admission. These professionals were instructed to complete the questionnaire following the same ordinal scale suggested above.

Table 4 shows the profile of the health professionals who participated as the target audience of this stage of material evaluation and validation. When analyzing the data obtained, we observed that from the total of 36 evaluators, 31 are female (86.1%) and 5 were male (13.9%). Regarding age group, there is a greater concentration of professionals and students in the 20 to 30 age group (63.9%), with a mean of 30.1 years and standard deviation (SD) of 7.17.

**Table 4:** Profile of the target audience that evaluated the screening protocol

**Variables**	**N**	**(%)**
**Gender**		
Female	31	(86.1)
Male	5	(13.9)
**Age**		
20 - 30 years old	23	(63.9)
31 - 40 years old	9	(25.0)
41 years old or more	4	(11.1)
**Graduation/Job**		
Medicine	7	(19.4)
Nursing	4	(11.1)
Nursing technician	15	(41.7)
University students	10	(27.8)

After evaluating the target audience, we performed a new quantitative analysis of the questionnaire, evaluating the proportions of the agreement described in Table 5. As there was no need for changes, due to CVI values - I above 0.78 for all items of the screening protocol, the appearance, and content of this stage were maintained.

**Table 5:** Dysphagia screening protocol content validation indexes - Target audience

**Assessment instrument items**	**Number of judges in agreement (n = 36)**	**CVI – I**
1 – Does it have dental elements?	36	1.00
2 – Do you use a dental prosthesis?	36	1.00
3 – Do you have any difficulty making movements with your face (Facial Paralysis)?	36	1.00
4 – Is it difficult to keep the food/liquid in the mouth? Does it escape or fall through the lips?	36	1.00
5 – Do you eat faster or slower than before the disease?	31	0.86
6 – Do you take a long time or find it difficult to eat solid foods after the illness?	35	0.97
7 – After you swallow, are there remains of food in your mouth?	35	0.97
8 - Do you feel that you have a lot of saliva in your mouth or drool frequently when you are awake?	33	0.92
9 – Do you feel pain when swallowing saliva, food, or liquids?	35	0.97
10 – Do you feel the food/liquid stuck in your throat?	35	0.97
11 – Do you need to swallow several times to help the food go down your throat?	36	1.00
12 – Did you start drinking liquids to help the food go down after the illness?	33	0.92
13 – Do you feel that your voice changes during or after the meal?	31	0.86
14 – Do you cough or clear your throat during a meal?	34	0.94
15 – Do you feel suffocated or have difficulty breathing when eating?	36	1.00
16 – Do you choke often?	36	1.00
	**CVI – T**	0.96

Thus, the protocol can be considered validated in its appearance and content, as it obtained a CVI – T of 0.92.

## DISCUSSION

From the articles selected for this study, we could create a multidisciplinary screening protocol, considering the main aspects related to the oral and pharyngeal phases of swallowing.

The validation of this protocol took place through the analysis by expert judges in HIV/AIDS as well as by the target audience that will apply it. The construction of validated educational materials is important to standardize behavior in the patient care, and the participation of all professionals is essential^(13)^.

Face validation is a measure that assesses the degree to which respondents consider the construction and content of a test and its items as relevant to the context in which the instrument will be applied. This means of validation is considered an important phase in the adaptation of questionnaires^(28)^ because it allows the assessment of the agreement of expert judges and the target audience on each item of a questionnaire and on the instrument as a whole. This method is the beginning of a process that should encompass other types of validation and reliability^(28)^.

When there is agreement among most raters, it is considered a robust construction of the research protocol. In this process, the participants' suggestions regarding the replacement of terms and reformulation of information are analyzed, improving the material^(16)^. In the data analysis, the Content Validity Index was used.

The experts for judging the instrument created in this study included professionals from the areas of Speech-Language Therapy, Nursing, Nutrition, and Medicine, with a minimum master's degree and at least three years of clinical experience in caring for patients with HIV. No official standard for the choice of judges was found in the literature.

In the validation by expert judges, the results showed that 85.7% of the sample consisted of female individuals. Regarding the age group, influenced by the inclusion criteria of the study, an average of 44.1 years old and an SD of 11.67 was found.

We chose experts from different areas of health given the multidisciplinary profile of the protocol, who work directly with the patient in clinical evaluations and therapy. All the invited professionals accepted to participate in the research. They were 3 speech-language therapists (42.8%), 2 nurses (28.6%), 1 physician (14.3%) and 1 nutritionist (14.3%). Regarding the length of academic training, most judges have up to 15 years (42.8%) of graduation.

Due to the minimum degree used as an inclusion criterion, there is little percentage difference between participants with a master's degree (57.1%) and a doctorate (42.9%). In the field of work, most judges (85.7%) are dedicated both to teaching and clinical care. Regarding bibliographic production, 5 of the evaluators (71.4%) have articles published in journals.

After a quantitative analysis of the judges' answers in the first phase, two items did not reach the CVI – I minimum of 0.78. They are: “6 – Do you take a long time, or do you find it difficult to eat hard foods?” (0.71) and “16 – Does the food or drink go to the “wrong place” when you swallow?” (0.71). The review of these items was carried out based on the qualitative analysis of the experts' suggestions.

The item “Do you take a long time, or do you find it difficult to eat hard foods?” was modified according to the suggestion of one of the experts to replace the nomenclature used “hard foods” with “solids”. We accepted the suggestion of another judge to use temporal words that indicate that the difficulty that the individual is experiencing is not before the disease but that it started to occur after his diagnosis.

We accepted the suggestion to change the way of describing the difficulty of the item “Does the food or drink go to the “wrong place” when you swallow?”, using then “Do you choke often?”, encompassing the frequency of this event occurs, so that it is considered a problem.

In addition to these mandatory changes, a survey of all the suggestions presented was also carried out, although with CVI – I considered within the appropriate standard, enabling improvements in the protocol created, through the heterogeneity of experiences of the participating judges. Therefore, we changed the words to make the questions more comprehensive and clearer. The words were also added that make the questions more self-explanatory and that represent the temporality of the question.

We also highlight the analysis of item 4: “Is it difficult to keep the food/liquid in the mouth? Does it escape or fall through the lips?”, for which the 7 judges marked the option “highly relevant”. This can be explained because neurological changes enable a decrease in muscle tone, which consequently causes greater difficulty in oral control of the bolus and the possibility of delay in the pharyngeal sequence of swallowing, providing a greater chance of premature escape of this bolus, both anterior and posterior and, risk of bronchoaspiration^(7)^. This difficulty is also often found in objective assessments of patients with neurological disorders^(29)^.

After these changes, the protocol was again sent to the expert judges for the follow-up of appearance and content validation.

In the quantitative analysis of the second phase of validation by the judges, with the modified protocol, a CVI – I above 0.78 was observed for all items, and a CVI – T of 0.92. Thus, it was possible to start the third phase of validation, with the assessment of the target audience.

It is also important to note that the question “Do you need to swallow several times to help the food go down from the throat?”, during this second assessment, received the 7 “highly relevant” ratings. This can be justified due to the change in the way the question was rewritten, enabling greater accessibility of the vocabulary to the reality of the study population. We can be found in the specialized literature that the sensation of bolus in the throat is closely related to the difficulty of swallowing seen in objective exams, leading to a higher risk for oropharyngeal dysphagia^(29)^.

The next phase of validation is extremely important since the target audience of the research was composed of individuals who normally have the first contact with the patient and who, therefore, will be responsible for applying the instrument. This population must be able to understand the questions that the questionnaire includes, as well as to reproduce them for the study population. This is the time to analyze opinions and suggestions for the preparation of this material, and to verify how the material was understood by these people and identify what was not clear^(13)^.

Regarding this target audience, the results showed that 86.1% of the sample consisted of female individuals. The age group average was 30.1 years old and had an SD of 7.17.

We had the evaluation of 10 university students from the last periods of different courses in the health sciences (27.8%), 15 professionals with technical training (41.7%), and 7 physicians (19.4%), and 4 nurses (11.1%).

After the quantitative analysis of this population, we observed that in the questions “5 - Do you eat faster or slower than before the disease?”, “6 - Do you take a long time or find it difficult to eat solid foods after the disease?”, “7 - After you swallow, are there still food residues in your mouth?”, “8 - Do you feel that you have a lot of saliva in your mouth or drool frequently when you are awake?”, “9 - Do you feel pain when swallowing saliva, food, or liquids?”, “10 - Do you feel the food/liquid stuck in your throat?”, “12 - Did you start drinking liquids to help the food go down after the illness?”, “13 - Do you feel that your voice changes during or after the meal?” and “14 - Do you cough or clear your throat during a meal?” at least 1 participant considered each of these questions to be of little relevance, but there was no need to change, as most evaluators classified the items as quite or highly relevant.

Slow oral transit time, difficulty in handling food, residues in the oral cavity, saliva stasis, odynophagia, feeling of stagnant food, wet voice, and cough^(29)^ are often symptoms found in individuals with feeding difficulties.

After the validation stage by the target audience, we observed through the quantitative analysis that there was no need to modify the questionnaire, due to CVI - I values above 0.78 for all items and CVI - T of 0.92. Thus, the protocol can be validated in its appearance and content, complying with its purpose.

We need further validation stages to be able to use the instrument in clinical practice.

## CONCLUSION

The multiprofessional dysphagia screening protocol in patients with HIV infection is a screening tool, which aims to identify early risk for difficulty in swallowing and interdiction of a possible pulmonary complication.

This protocol can be considered validated from the point of view of appearance and content since it presented a total CVI of 0.92 in the expert judges' validation and a total CVI of 0.96 from the validation by the target audience. Thus, we verified a protocol CVI of 0.94. Therefore, we can consider the resolution instrument capable of fulfilling its proposals.

Because of the suggestions and contributions of all participants during the validation process, the protocol was changed in its language and structure, making it more comprehensive and qualified for its use in the day-to-day of people who work with these patients covered in the research.

The protocol has been validated in its content and appearance. Other studies are needed to verify its use, including large-scale experimental applications with coverage of different professionals and institutions (internal and external validation and reliability) so that the instrument can be used in clinical practice.

## Supplementary Material

Supplementary material accompanies this paper.

Appendix 1 Dysphagia Screening Protocol in HIV Patients

Appendix 2 Appearance and Content Validation Form

This material is available as part of the online article from https://www.scielo.br/j/codas
