# Supplementary material for: Multiprofessional screening protocol for dysphagia in patients with HIV infection: elaboration and content validity
Source: Codas. 2021 Oct 22;34(2):e20210012. doi: 10.1590/2317-1782/20212021012 (PMC9769427; doi:10.1590/2317-1782/20212021012)
Supplement: Appendix 1 [file codas-34-2-e20210012-app.pdf]

Appendix 1 – Screening Protocol for Dysphagia in Patients with HIV Infection

Name: ___________________________________ Age: ________ Date: ___________ Sex ( ) M ( ) F
Time since diagnosis: __________ Medical Record: ___________ CD4: ________ TARV use ( ) Y ( ) N
Estimated weight: ___________ Image/ instrumental exams: _________________________________
History of Pneumonia ( ) Y ( ) N Reflux/ Heartburn ( ) Y ( ) N Change in voice/ speech ( ) Y ( ) N
Professional:( ) Doctor ( ) Nurse/ Nursing Technician ( ) Speech Therapist ( ) Other _________

Clinical criteria:

- ☐ Tracheostomized patient
- ☐ Patient cannot remain alert
- ☐ Does not remain seated/postured
- ☐ Need oxygen support
- ☐ Use of alternative feeding route
- ☐ Oral infections by Candida
- ☐ Complaints to feed (needs special preparation or thickened liquid)

→ If you have checked any of the options refer to the speech therapist evaluation

→ If you have not checked any of the options, start the questionnaire

1 – Do you have a complete dentition? ( ) Y ( ) N
( ) complete ( ) incomplete

2 – Do you use a dental prosthesis? ( ) S ( ) N
( ) top ( ) well adapted ( ) wide
( ) down ( ) well adapted ( ) wide

3 – Do you have any difficulty making movements with your face (Facial Paralysis)?
( ) Y ( ) N

4 – Is it difficult to keep the food/liquid in the mouth? Does it escape or fall through the lips?
( ) Y ( ) N

5 – Do you feed faster or slower than before the illness?
( ) Y ( ) N

6 – Do you take more time or find it difficult to eat solid foods after the illness?
( ) Y ( ) N

7- Is there still food left in your mouth after you swallow?
( ) Y ( ) N

8 – Do you feel that you have a lot of saliva in your mouth or drool frequently when you are awake?
( ) Y ( ) N

9 – Do you feel pain when swallowing saliva, food or liquids?
( ) Y ( ) N

10 – Do you feel the food/liquid stuck in your throat?
( ) Y ( ) N

11 – Do you need to swallow several times to help the food go down your throat?
( ) Y ( ) N

12 – Did you start drinking fluids to help the food go down after the illness?
( ) Y ( ) N

13 – Do you feel your voice changing during or after a meal?
( ) Y ( ) N

14 – Do you cough or clear your throat during a meal?
( ) Y ( ) N

15 – Do you feel suffocated or have difficulty breathing while eating?
( ) Y ( ) N

16 – Do you choke often?
( ) Y ( ) N

Appendix 2 – Appearance and Content Validation Form

1 – Do you have a complete dentition? ( ) Y ( ) N
( ) complete ( ) incomplete
( ) NR ( ) LR ( ) QR ( ) HR

2 – Do you use a dental prosthesis? ( ) Y ( ) N
( ) top ( ) well adapted ( ) wide
( ) down ( ) well adapted ( ) wide
( ) NR ( ) LR ( ) QR ( ) HR

3 – Do you have any difficulty making movements with your face (Facial Paralysis)?
( ) Y ( ) N
( ) NR ( ) LR ( ) QR ( ) HR

4 – Is it difficult to keep the food/liquid in the mouth? Does it escape or fall through the lips?
( ) Y ( ) N
( ) NR ( ) LR ( ) QR ( ) HR

5 – Do you feed faster or slower than before the illness?
( ) Y ( ) N
( ) NR ( ) LR ( ) QR ( ) HR

6 – Do you take more time or find it difficult to eat solid foods after the illness?
( ) Y ( ) N
( ) NR ( ) LR ( ) QR ( ) HR

7- Is there still food left in your mouth after you swallow?
( ) Y ( ) N
( ) NR ( ) LR ( ) QR ( ) HR

8 – Do you feel that you have a lot of saliva in your mouth or drool frequently when you are awake?
( ) Y ( ) N
( ) NR ( ) LR ( ) QR ( ) HR

9 – Do you feel pain when swallowing saliva, food or liquids?
( ) Y ( ) N
( ) NR ( ) LR ( ) QR ( ) HR

10 – Do you feel the food/liquid stuck in your throat?
( ) Y ( ) N
( ) NR ( ) LR ( ) QR ( ) HR

11 – Do you need to swallow several times to help the food go down your throat?
( ) Y ( ) N
( ) NR ( ) LR ( ) QR ( ) HR

12 – Did you start drinking fluids to help the food go down after the illness?
( ) Y ( ) N
( ) NR ( ) LR ( ) QR ( ) HR

13 – Do you feel your voice changing during or after a meal?
( ) Y ( ) N
( ) NR ( ) LR ( ) QR ( ) HR

14 – Do you cough or clear your throat during a meal?
( ) Y ( ) N
( ) NR ( ) LR ( ) QR ( ) HR

15 –Do you feel suffocated or have difficulty breathing while eating?
( ) Y ( ) N
( ) NR ( ) LR ( ) QR ( ) HR

16 – Do you choke often?
( ) Y ( ) N
( ) NR ( ) LR ( ) QR ( ) HR

Subtitle:
NR - Not relevant/ **LR -** Little relevant**/ QR** - Quite relevant/ H**R -** Highly relevant

Apêndice 1 – Protocolo de Rastreio de Disfagia em Pacientes HIV

Nome: ______________________________ Idade: ________ Data: ___________ Sexo ( ) M ( ) F
Tempo de diagnóstico: __________ Prontuário: ___________ CD4: ________ Uso de TARV ( ) S ( ) N
Peso estimado: ___________ Exames de imagem/instrumental: ________________________________
Histórico de Pneumonia ( ) S ( ) N Refluxo/ Azia ( ) S ( ) N Alteração na voz/fala ( ) S ( ) N
Profissional:( ) Médico ( ) Enfermeiro/Técnico de Enfermagem ( ) Fonoaudiólogo ( ) Outro _________

Critérios clínicos:

- ☐ Paciente traqueostomizado
- ☐ Paciente não consegue manter-se alerta
- ☐ Não permanece sentado/posturado
- ☐ Necessita de suporte de oxigênio
- ☐ Uso de via alternativa de alimentação
- ☐ Presença de candidíase oral
- ☐ Queixas para se alimentar (necessita de preparo especial ou líquidos espessados)

→ Caso tenha marcado alguma das opções encaminhar para avaliação Fonoaudiológica

→ Caso não tenha marcado nenhuma das opções, iniciar o questionário

1 – Possui elementos dentários? ( ) S ( ) N
( ) completos ( ) incompletos

2 – Faz uso de prótese dentária? ( ) S ( ) N
( ) superior ( ) bem adaptada ( ) frouxa
( ) inferior ( ) bem adaptada ( ) frouxa

3 – Possui alguma dificuldade para fazer movimentos com o rosto (Paralisia Facial)?
( ) S ( ) N

4 – Há dificuldade de manter a comida/líquido dentro da boca? Escapa ou cai pelos lábios?
( ) S ( ) N

5 – Você se alimenta de forma mais rápida ou mais lenta do que antes da doença?
( ) S ( ) N

6 – Você passou a demorar ou ter dificuldades para comer alimentos sólidos depois da doença?
( ) S ( ) N

7- Depois que você engole ainda ficam restos de alimento na boca?
( ) S ( ) N

8 – Você sente que tem muita saliva na boca ou baba com frequência quando está acordado?
( ) S ( ) N

9 – Você sente dor para engolir saliva, alimentos ou líquidos?
( ) S ( ) N

10 – Sente o alimento/líquido preso na garganta?
( ) S ( ) N

11 – Precisa engolir várias vezes para ajudar o alimento a descer da garganta?
( ) S ( ) N

12 – Passou a beber líquidos para ajudar a comida a descer depois da doença?
( ) S ( ) N

13 – Sente que sua voz muda durante ou depois da refeição?
( ) S ( ) N

14 – Você tosse ou pigarreia durante a refeição?
( ) S ( ) N

15 – Se sente sufocado ou com dificuldade para respirar ao se alimentar?
( ) S ( ) N

16 – Você engasga frequentemente?
( ) S ( ) N

Apêndice 2 – Formulário para Validação da Aparência e Conteúdo

1 – Possui elementos dentários? ( ) S ( ) N
( ) completos ( ) incompletos
( ) NR ( ) PR ( ) BR ( ) AR

2 – Faz uso de prótese dentária? ( ) S ( ) N
( ) superior ( ) bem adaptada ( ) frouxa
( ) inferior ( ) bem adaptada ( ) frouxa
( ) NR ( ) PR ( ) BR ( ) AR

3 – Possui alguma dificuldade para fazer movimentos com o rosto (Paralisia Facial)?
( ) S ( ) N
( ) NR ( ) PR ( ) BR ( ) AR

4 – Há dificuldade de manter a comida/líquido dentro da boca? Escapa ou cai pelos lábios?
( ) S ( ) N
( ) NR ( ) PR ( ) BR ( ) AR

5 – Você se alimenta de forma mais rápida ou mais lenta do que antes da doença?
( ) S ( ) N
( ) NR ( ) PR ( ) BR ( ) AR

6 – Você passou a demorar ou ter dificuldades para comer alimentos sólidos depois da doença?
( ) S ( ) N
( ) NR ( ) PR ( ) BR ( ) AR

7- Depois que você engole ainda ficam restos de alimento na boca?
( ) S ( ) N
( ) NR ( ) PR ( ) BR ( ) AR

8 – Você sente que tem muita saliva na boca ou baba com frequência quando está acordado?
( ) S ( ) N
( ) NR ( ) PR ( ) BR ( ) AR

9 – Você sente dor para engolir saliva, alimentos ou líquidos?
( ) S ( ) N
( ) NR ( ) PR ( ) BR ( ) AR

10 – Sente o alimento/líquido preso na garganta?
( ) S ( ) N
( ) NR ( ) PR ( ) BR ( ) AR

11 – Precisa engolir várias vezes para ajudar o alimento a descer da garganta?
( ) S ( ) N
( ) NR ( ) PR ( ) BR ( ) AR

12 – Passou a beber líquidos para ajudar a comida a descer depois da doença?
( ) S ( ) N
( ) NR ( ) PR ( ) BR ( ) AR

13 – Sente que sua voz muda durante ou depois da refeição?
( ) S ( ) N
( ) NR ( ) PR ( ) BR ( ) AR

14 – Você tosse ou pigarreia durante a refeição?
( ) S ( ) N
( ) NR ( ) PR ( ) BR ( ) AR

15 –Se sente sufocado ou com dificuldade para respirar ao se alimentar?
( ) S ( ) N
( ) NR ( ) PR ( ) BR ( ) AR

16 – Você engasga frequentemente?
( ) S ( ) N
( ) NR ( ) PR ( ) BR ( ) AR

Legenda:
NR - Não relevante/ **PR -** Pouco relevante**/ BR -** Bastante relevante/ **AR -** Altamente relevante
