# Supplementary material for: Multiprofessional screening protocol for dysphagia in patients with HIV infection: elaboration and content validity
Source: Codas. 2021 Oct 22;34(2):e20210012. doi: 10.1590/2317-1782/20212021012 (PMC9769427; doi:10.1590/2317-1782/20212021012)
Supplement: Translation

# Translation: Protocolo de rastreio multiprofissional de disfagia em pacientes com infecção HIV: elaboração e validação de conteúdo

**Authors:** Luise Alexandre Rocha Soutinho, Daniel Aragão Machado, Charles Henrique Dias Marques

**Affiliations:** 1 Universidade Federal do Rio de Janeiro – UFRJ - Rio de Janeiro (RJ), Brasil.; 2 Universidade Federal do Estado do Rio de Janeiro – UNIRIO - Rio de Janeiro (RJ), Brasil.

**Keywords:** Transtornos de Deglutição, Protocolos, Programas de Rastreamento, Síndrome da Imunodeficiência Adquirida, Infecções por HIV

## Abstract

**Objetivo:**

Elaborar e validar um protocolo multiprofissional para identificação do risco de disfagia em pessoas com HIV no momento da internação hospitalar.

**Método:**

Após revisão bibliográfica, o protocolo de rastreio de disfagia criado foi submetido a análise de juízes especialistas em HIV/Aids e público-alvo na aplicação deste instrumento para realização da validação de face/conteúdo. Esses avaliadores poderiam sugerir mudanças no protocolo, julgando os quesitos como clareza, pertinência e abrangência. Para avaliar o grau de concordância do instrumento, foi utilizado o Índice de Validade de Conteúdo (IVC) mínimo de 0,78.

**Resultados:**

O protocolo foi elaborado com 16 itens, incluindo aspectos relacionados à fase oral e faríngea da deglutição, e a pontuação final foi calculada baseada nos diferentes riscos para complicações clínicas. O instrumento apresentou IVC acima de 0,78 para todos os itens nas duas fases da validação, bem como IVC total de 0,92.

**Conclusão:**

A partir dos dados obtidos, foi possível a construção e validação do protocolo de rastreio do ponto de vista de aparência e conteúdo, uma vez que apresentou IVC total acima do valor mínimo estipulado na validação dos juízes especialistas e do público-alvo, constatando-se assim IVC adequado para o protocolo como um todo.

## INTRODUÇÃO

A Síndrome da Imunodeficiência Adquirida (SIDA-Aids) teve seus primeiros casos registrados nos Estados Unidos, Haiti e África Central no final da década de 1970, sendo uma infecção causada pelo vírus da imunodeficiência humana, HIV, que afeta as células do sistema imunológico^(1)^.

Desde a descoberta dos primeiros casos dessa infecção até o final dos anos 1990, estima-se, segundo a Organização Mundial de Saúde (OMS), que cerca de 29,4 milhões de pessoas tenham sido infectadas pelo HIV^(2)^.

No Brasil, os primeiros casos só foram identificados na década de 80, sendo, somente em 1983, estruturado o primeiro programa de controle da Aids no país^(3)^. De acordo com o boletim epidemiológico, nos primeiros 30 anos foram notificados no SINAN (Sistema de Informação de Agravos de Notificação) mais de 650 mil casos em que a Aids se manifestou^(4)^.

A infecção pelo vírus resulta em ampla variação de manifestações clínicas, podendo encontrar desde indivíduos assintomáticos, até sintomas como fraqueza, febre, emagrecimento e diarreia prolongada, candidíase, neurotoxoplasmose, entre outros^(5)^. Na evolução da infecção pelo HIV, o vírus entra no sistema nervoso central (SNC) podendo causar transtornos da função cognitiva, levando a déficits em processos como atenção e memória, além de demência e sintomas motores^(6)^.

As manifestações em região orofaríngea, sendo a candidíase a causa mais comum, podem apresentar sintomas como dor oral, gosto amargo ou azedo na boca ou até esofagite com odinofagia (dor ao deglutir)^(5)^. Como consequência desses sintomas, podemos inferir que esse indivíduo pode apresentar desde transtornos na articulação da fala, devido às lesões, até dificuldades de deglutição (disfagia).

Deglutir é atividade neuromuscular que transporta o alimento da cavidade oral para o estômago, envolvendo a coordenação de estruturas que participam tanto da deglutição quanto da respiração, não permitindo a entrada de substâncias nas vias respiratórias^(7)^. A disfagia é um distúrbio durante esse processo, sendo definida como um sintoma, em que a alteração da deglutição é decorrente de alguma doença base.

Embora as fases da deglutição sejam divididas didaticamente em fase oral, faríngea e esofágica, deglutir é um processo contínuo; por isso, qualquer comprometimento em apenas uma dessas fases pode influenciar nas demais, levando ao prejuízo em todo o processo.

À medida que a infecção pelo HIV progride em indivíduos contaminados, pode ocorrer redução gradativa na massa corporal magra que, por sua vez, associa-se à redução da capacidade do sistema musculoesquelético em gerar força^(8)^. Consequentemente, o desempenho funcional das estruturas relacionadas à deglutição é afetado, já que a deglutição é uma função exercida por esforço. Além dessa hipotonia, a microbiota bucal de pacientes HIV positivos é diferente (apresentam aumento de leveduras de várias espécies) da microbiota bucal dos indivíduos imunocompetentes^(1)^, o que, associado à baixa imunidade do paciente, leva ao aparecimento de manifestações bucais, que estão intimamente relacionadas a comprometimentos significativos na fase oral da deglutição^(9)^.

Em revisão bibliográfica é possível encontrar um estudo que relata sintomas de disfagia em 47% dos pacientes com Aids^(10)^. Esses prejuízos relacionados à fase oral da deglutição influenciam não só na ejeção do bolo alimentar, mas também na sequencialidade do processo. Quando existe acometimento esofágico, essa incidência sobe para 59 a 79% dos pacientes^(4)^.

O cuidado multiprofissional é imprescindível para indivíduos com disfagia, levando-se em consideração que as consequências dessa dificuldade envolvem aspectos clínicos, além de possíveis impactos na qualidade de vida e nos aspectos sociais da alimentação, já que pode levar à retração e isolamento do indivíduo.

Na avaliação da deglutição, torna-se necessário o conhecimento de como algumas doenças, manifestadas no indivíduo portador de HIV, influenciam na função de deglutição e suas fases. A elaboração de protocolos que norteiem a ação da equipe multiprofissional visa garantir a qualidade do serviço oferecido, além de definição de condutas a serem tomadas.

Em estudo prévio, observou-se uma redução da incidência de pneumonia aspirativa em pacientes hospitalizados, a partir da aplicação de protocolo formal de avaliação da disfagia^(11)^. A identificação precoce da dificuldade e o encaminhamento antecipado para o Fonoaudiólogo visam evitar episódios de broncoaspiração e, consequentemente, reduzir o tempo de internação hospitalar e os riscos de infecção, promovendo diminuição dos gastos do sistema de saúde e proporcionando melhor qualidade de vida para esses sujeitos.

O presente estudo propôs criar um protocolo multiprofissional para identificação do risco de disfagia em pessoas que vivem com HIV no momento da internação hospitalar. Além disso, tem o objetivo de validar o protocolo, junto a especialistas no atendimento de pessoas que vivem com HIV e junto à equipe multiprofissional, com vias à validação de concordância do público-alvo.

## MÉTODO

Trata-se de estudo descritivo, com abordagem quantitativa, que visa à criação de um produto, bem como sua validação, de modo a torná-lo confiável e válido para o fim que se destina.

O instrumento criado neste estudo consiste em um questionário a ser validado e aplicado por diversos profissionais de saúde, especificamente em pacientes com diagnóstico de HIV, com idade mínima de 18 anos, no momento de sua internação hospitalar.

O estudo foi dividido em duas etapas: a criação do questionário e sua validação baseada no conteúdo.

A primeira etapa foi a realização de uma revisão de literatura sobre o uso de protocolos de rastreio de disfagia e métodos de validação de questionários. Esta revisão permitiu analisar os protocolos já validados para adaptação de suas perguntas e criação do novo protocolo específico para pessoas que vivem com HIV.

Para essa revisão de literatura, foram feitas buscas avançadas nas bases de dados BVS e PUBMED. Quanto à temporalidade, foram incluídos estudos publicados entre 1984 e 2015, utilizando os descritores “acquired immunodeficiency syndrome”, “deglutition disorders”, “protocols”, “triage” e “mass screening”, além de “face validity”, suas combinações e suas respectivas traduções para a língua portuguesa. Além disso, as revisões e as listas de referências de todos os artigos considerados relevantes foram consultadas em busca de novos artigos para inclusão. A busca foi realizada por meio de palavras encontradas nos títulos, nos resumos e no corpo do texto.

A elaboração do protocolo foi realizada pelos autores do trabalho, pesquisadores que apresentam experiência na área de disfagia e vivência com pacientes com HIV.

Ao final desta primeira etapa foi desenvolvido o protocolo intitulado como: “Protocolo Multiprofissional de Rastreio de Disfagia em Pacientes HIV” (Apêndice 1), que contemplou aspectos como: critérios clínicos de risco para disfagia orofaríngea e alterações da fase oral e faríngea da deglutição.

Após essa fase, iniciou-se a segunda etapa da pesquisa, na qual foi utilizada a Validação de Face/Conteúdo por ser um método comum na área da saúde^(12)^. É importante que esse processo seja feito por profissionais de diferentes áreas e que sejam realmente peritos na temática do estudo, valorizando as diferentes sugestões e opiniões sobre o tema^(13)^.

Para esta fase da validação de conteúdo foram seguidas as seguintes orientações: escolha dos juízes especialistas no assunto e suas avaliações quanto aos itens individuais e do questionário como um todo e escolha e avaliação do público-alvo (profissionais que aplicarão o questionário na prática clínica). Todas as avaliações foram feitas através do Índice de Validade de Conteúdo.

Quanto ao número ideal de juízes para o processo de validação, não há na literatura um consenso, devendo-se levar em conta as características do instrumento^(14)^. Sendo assim, para este estudo, foram utilizados sete juízes de diferentes áreas da saúde, que contemplassem os seguintes critérios de inclusão: curso mínimo de mestrado em suas áreas e pelo menos três anos de experiência clínica em cuidados com pacientes com HIV.

Cada um desses profissionais convidados a participar do estudo recebeu: carta convite com os objetivos do trabalho, protocolo de rastreio (Apêndice 1) e formulário para validação da aparência e conteúdo (Apêndice 2). A avaliação do protocolo pôde ser realizada em local de preferência de cada avaliador, sendo estabelecido um prazo de quatorze dias para devolução do instrumento preenchido.

Os juízes escolhidos foram orientados a avaliar cada um dos 16 itens do questionário criado para o estudo, com relação aos seguintes critérios: clareza da linguagem escrita utilizada, pertinência do assunto em questão e abrangência dos aspectos referentes ao tema abordado. Além disso, para todos os itens avaliados foi dada possibilidade de incluir sugestões. A coleta de dados foi feita no último semestre de 2017.

Após avaliação e possível modificação do protocolo pelos especialistas, houve o julgamento do material pelo público-alvo, ou seja, avaliação quanto à aparência, linguagem e aplicabilidade pelos profissionais que utilizarão esta ferramenta em indivíduos com HIV. É um momento importante para o estudo, visto que é possível verificar a compreensão da população que irá aplicar o protocolo, bem como analisar suas sugestões. A etapa de análise de aparência tem o objetivo de averiguar se o material é compreensível à população à qual se destina.

É indicada a escolha de 30 a 40 pessoas da população alvo para avaliação desta etapa do estudo^(15)^. Dentre esses profissionais, foram selecionados diferentes componentes da equipe de saúde, visando garantir a característica multiprofissional do protocolo desenvolvido.

A amostra do público-alvo se deu por conveniência e foi composta por 36 profissionais de saúde lotados em um hospital universitário, seguindo alguns critérios de inclusão: ter idade igual ou superior a 18 anos; ter disponibilidade de tempo para participar de forma voluntária na avaliação do protocolo de rastreio e possuir vínculo de trabalho ou estudantil com o hospital em estudo.

Novamente, para todos que aceitaram participar da pesquisa, foi entregue: protocolo de rastreio (Apêndice 1) e formulário para validação da aparência e conteúdo (Apêndice 2). Além disso, foi solicitada a assinatura do Termo de Consentimento Livre e Esclarecido - TCLE.

O público abordado foi orientado a avaliar cada um dos 16 itens do protocolo de rastreio multiprofissional de disfagia, com relação aos mesmos critérios de clareza da linguagem escrita, pertinência e abrangência do assunto em questão. A avaliação do protocolo foi realizada em local à escolha dos participantes, sendo estabelecido prazo de um dia para devolução do instrumento preenchido.

Assim como os juízes especialistas, o público-alvo recebeu as orientações quanto ao preenchimento do questionário em diferentes momentos, porém feito pelo mesmo indivíduo, procurando manter linguagem e instrução de forma equânime, objetivando reduzir possíveis distorções.

O Índice de Validade de Conteúdo – IVC é destinado a avaliar a porcentagem de juízes que estão em concordância sobre determinado item. É considerado válido se, após a análise das respostas dos juízes, obtiver índice de aprovação acima de 78% (0,78)^(16)^. Para se calcular o IVC para cada item do questionário (IVC – I), foi dividido o número total de juízes que atribuiu escore de 3 ou 4 em uma escala ordinal de quatro pontos com significância de “irrelevante” a “extremamente relevante” pelo total de juízes que participaram da avaliação^(16)^.

Para avaliar o instrumento como um todo (IVC – T), foi utilizada a média do número total de itens considerados como relevantes pelos juízes, pelo número total de itens no questionário. Foi aceito para aprovação IVC – T acima de 90% (0,90)^(16)^.

As avaliações inferiores a estes valores, tanto para cada item quanto para o valor total do teste, foram revisadas e reenviadas aos mesmos juízes, visando que todos alcançassem o valor mínimo de confiabilidade.

Essa etapa faz parte de um processo amplo de validação do instrumento. Nesse sentido, essa fase abrange a criação e a aceitação das perguntas utilizadas no questionário por pessoas especialistas no assunto, bem como por profissionais da saúde que aplicarão o protocolo na prática clínica. Somente após essa etapa, será possível utilizar o instrumento com os indivíduos portadores de HIV.

O presente projeto foi submetido e aprovado pelo Comitê de Ética em Pesquisa da Universidade Federal do Estado do Rio de Janeiro - UNIRIO em 30 de agosto de 2017, sob o parecer de número 2.247.883.

Todos os aspectos éticos pertinentes à pesquisa e à participação no estudo foram descritos no TCLE, que foi assinado após aceitação dos participantes. Além disso, todos foram orientados sobre a privacidade dos dados da pesquisa, também da garantia de que as informações obtidas não poderiam ser usadas para outros fins além dos previstos no projeto, e da possibilidade de recusar ou interromper sua participação a qualquer momento, sem qualquer tipo de prejuízo ou penalização.

## RESULTADOS

O protocolo multiprofissional de rastreio de disfagia em pacientes HIV foi elaborado pelos pesquisadores do estudo com base no conteúdo de artigos presentes na literatura^(17-27)^, além da experiência dos autores para formulação da divisão dos itens e pontuação atribuída a eles.

Foram levantados 21 artigos, sendo 3 em português e 18 em inglês. Desses, 16 possuíam questionários validados de avaliação de disfagia, sendo apenas 11 com perguntas pertinentes ao estudo e 5 referentes a métodos de validação. Com base nesses artigos, o protocolo de rastreio foi criado levando em consideração aspectos presentes na dinâmica da deglutição.

Foi de suma importância transformar a linguagem das informações localizadas na literatura científica e específica em uma linguagem acessível ao público-alvo^(13)^; por isso, todos os termos técnicos da área de Fonoaudiologia foram substituídos por sinônimos que alcançassem o significado da informação.

Os textos foram escritos utilizando-se estilo de letras simples e de fácil leitura, fonte *Arial* tamanho 10 para as informações e 12 para o título. Além disso, foram utilizados elementos gráficos para comunicar informações visualmente, de maneira que retratassem um passo a passo de triagem capaz de localizar os principais fatores de risco para disfagia e encaminhamento para o profissional capacitado, além de setas destacando informações-chave durante a aplicação do protocolo.

O protocolo de rastreio foi dividido em três partes:

1. Perfil do paciente: apresenta dados como identificação do sujeito, dados de exames anteriores, tempo de diagnóstico, uso de medicação antirretroviral, peso, queixas, história patológica pregressa e identificação do profissional que está aplicando o questionário;
2. Critérios clínicos de risco para disfagia: indica presença de traqueostomia, necessidade de suporte de oxigênio, dificuldade de manter nível de alerta ou postura adequada para alimentação, presença de lesões em cavidade oral ou queixas que representem um risco elevado para disfagia orofaríngea;
3. Sinais e sintomas: contempla 16 questões criadas para este estudo que podem representar alguma dificuldade na fase oral e faríngea da deglutição, levando este indivíduo a um maior risco de apresentar disfagia.

É importante salientar que foi considerado ser interrompido o preenchimento do protocolo quando assinalado qualquer item de critérios clínicos, já que leva o indivíduo a um alto risco para disfagia, sendo necessária avaliação fonoaudiológica precoce.

A forma final do protocolo foi intitulada como: “Protocolo Multiprofissional de Rastreio de Disfagia em Pacientes HIV” e sua primeira versão foi enviada aos juízes especialistas. Para essa análise, foram escolhidos profissionais da área de Fonoaudiologia, Enfermagem, Nutrição e Medicina que tenham aceitado participar da pesquisa.

A Tabela 1 apresenta o perfil dos 7 juízes especialistas selecionados e que validaram o material de estudo. De acordo com a tabela, percebe-se que, do total de juízes, 6 são do gênero feminino (85,7%) e 1 do masculino (14,3%). Com relação à faixa etária, observamos que há maior concentração de especialistas na faixa de 30 a 40 anos (57,1%), sendo a média de 44,1 anos e desvio padrão (DP) de 11,67.

**Tabela 1:** Perfil dos juízes especialistas avaliadores do protocolo de rastreio

**Variáveis**	**N**	**(%)**
**Sexo**		
Feminino	6	(85,7)
Masculino	1	(14,3)
**Idade**		
30 - 40 anos	4	(57,1)
41 - 50 anos	1	(14,3)
51 anos ou mais	2	(28,6)
**Formação**		
Fonoaudiologia	3	(42,8)
Enfermagem	2	(28,6)
Medicina	1	(14,3)
Nutrição	1	(14,3)
**Tempo de Formação**		
Até 15 anos	3	(42,8)
16 – 30 anos	2	(28,6)
31 anos ou mais	2	(28,6)
**Titulação**		
Mestrado	4	(57,1)
Doutorado	3	(42,9)
**Área de trabalho**		
Docência	--	--
Assistência	1	(14,3)
Docência e Assistência	6	(85,7)
**Artigos Publicados**		
Sim	5	(71,4)
Não	2	(28,6)

Para cada questão, se seguia uma escala ordinal composta por: “não relevante” (NR), “pouco relevante” (PR), “bastante relevante” (BR) e “altamente relevante” (AR), que deveria ser assinalada conforme os critérios estabelecidos anteriormente.

Na primeira parte da validação, todos os juízes devolveram os questionários preenchidos dentro do prazo, os quais foram analisados quantitativamente, observando-se as proporções de concordância descrita na Tabela 2.

**Tabela 2:** Índices de validação de conteúdo do protocolo de rastreio de disfagia - Juízes - 1ª fase

**Itens do instrumento de avaliação**	**Número de juízes em concordância (n = 7)**	**IVC – I**
1 – Possui elementos dentários?	6	0,86
2 – Faz uso de prótese dentária?	6	0,86
3 – Possui alguma dificuldade para fazer movimentos com o rosto?	6	0,86
4 – Há dificuldade de manter a comida/ líquido dentro da boca? Escapa ou cai pelos lábios?	7	1,00
5 – Você leva mais tempo para se alimentar do que antigamente?	6	0,86
6 – Você demora ou tem dificuldade para comer alimentos duros?	5	0,71*
7 – Depois que você engole ainda ficam restos de alimento na boca?	6	0,86
8 - Você sente que tem muita saliva na boca ou baba com frequência?	7	1,00
9 – Você sente dor para engolir saliva, alimentos ou líquidos?	7	1,00
10 – Sente o alimento/ líquido preso na garganta?	7	1,00
11 – Você precisa engolir várias vezes para sentir que o alimento saiu da garganta?	7	1,00
12 – Precisa beber líquidos para ajudar a comida a descer?	6	0,86
13 – Sente que sua voz muda durante ou depois da refeição?	6	0,86
14 – Você tosse ou pigarreia durante a refeição?	6	0,86
15 – Se sente sufocado ou com dificuldade para respirar ao se alimentar?	7	1,00
16 – A comida ou bebida vai para o “lugar errado” quando você engole?	5	0,71*
	**IVC – T**	0,89

* Itens que obtiveram CVI – I inferior a 0,78

Após análise e modificações pertinentes, o protocolo, em sua 2ª versão (Apêndice 1), foi novamente enviado aos juízes especialistas para que julgassem o material, agora modificado, dando seguimento à validação de aparência e conteúdo.

A Tabela 3 mostra a análise quantitativa da concordância entre os juízes e a análise qualitativa das sugestões apontadas, agora com o protocolo modificado.

**Tabela 3:** Índices de validação de conteúdo do protocolo de rastreio de disfagia – Juízes - 2ª fase

**Itens do instrumento de avaliação**	**Número de juízes em concordância (n = 7)**	**IVC – I**
1 – Possui elementos dentários?	6	0,86
2 – Faz uso de prótese dentária?	6	0,86
3 – Possui alguma dificuldade para fazer movimentos com o rosto (Paralisia Facial)?	6	0,86
4 – Há dificuldade de manter a comida/ líquido dentro da boca? Escapa ou cai pelos lábios?	7	1,00
5 – Você se alimenta de forma mais rápida ou mais lenta do que antes da doença?	6	0,86
6 – Você passou a demorar ou ter dificuldade para comer alimentos sólidos depois da doença?	6	0,86
7 – Depois que você engole ainda ficam restos de alimento na boca?	7	1,00
8 - Você sente que tem muita saliva na boca ou baba com frequência quando está acordado?	6	0,86
9 – Você sente dor para engolir saliva, alimentos ou líquidos?	7	1,00
10 – Sente o alimento/ líquido preso na garganta?	7	1,00
11 – Precisa engolir várias vezes para ajudar o alimento a descer da garganta?	7	1,00
12 – Passou a beber líquidos para ajudar a comida a descer depois da doença?	6	0,86
13 – Sente que sua voz muda durante ou depois da refeição?	6	0,86
14 – Você tosse ou pigarreia durante a refeição?	6	0,86
15 – Se sente sufocado ou com dificuldade para respirar ao se alimentar?	7	1,00
16 – Você engasga frequentemente?	7	1,00
	**IVC – T**	0,92

Após essa segunda fase específica de validação pelos juízes, a análise quantitativa mostrou IVC – I acima de 0,78 para todos os itens modificados do protocolo de rastreio. Sendo assim, pode-se considerar o protocolo parcialmente validado, já que obteve IVC – T de 0,92, estando apto para iniciar a terceira fase da validação, que consiste na avaliação do público-alvo.

O público-alvo para avaliação do protocolo foi composto por profissionais da área de Medicina e Enfermagem, além de Técnicos e universitários dos cursos citados, que atuassem nos primeiros cuidados do paciente com perfil da pesquisa, durante sua admissão hospitalar. Esses profissionais foram orientados quanto ao preenchimento do questionário seguindo a mesma escala ordinal sugerida anteriormente.

A Tabela 4 apresenta o perfil dos profissionais de saúde que participaram como público-alvo dessa etapa da avaliação e validação do material. Ao analisar os dados obtidos, observa-se que do total de 36 avaliadores, 31 são do gênero feminino (86,1%) e 5 do masculino (13,9%). Em relação à faixa etária, verifica-se que há maior concentração de profissionais e estudantes na faixa de 20 a 30 anos (63,9%), sendo a média de 30,1 anos e desvio padrão (DP) de 7,17.

**Tabela 4:** Perfil do público-alvo que avaliou o protocolo de rastreio

**Variáveis**	**N**	**(%)**
**Sexo**		
Feminino	31	(86,1)
Masculino	5	(13,9)
**Idade**		
20-30 anos	23	(63,9)
31-40 anos	9	(25,0)
41 anos ou mais	4	(11,1)
**Formação/ Atuação**		
Medicina	7	(19,4)
Enfermagem	4	(11,1)
Téc. de enfermagem	15	(41,7)
Universitários	10	(27,8)

Após a avaliação do público-alvo, foi feita nova análise quantitativa do questionário, avaliando-se as proporções de concordância descrita na Tabela 5. Como não houve necessidade de modificações, devido aos valores de IVC – I acima de 0,78 para todos os itens do protocolo de rastreio, foi mantida a aparência e conteúdo desta etapa.

**Tabela 5:** Índices de validação de conteúdo do protocolo de rastreio de disfagia – Público-alvo

**Itens do instrumento de avaliação**	**Número de juízes em concordância (n = 36)**	**IVC – I**
1 – Possui elementos dentários?	36	1,00
2 – Faz uso de prótese dentária?	36	1,00
3 – Possui alguma dificuldade para fazer movimentos com o rosto (Paralisia Facial)?	36	1,00
4 – Há dificuldade de manter a comida/ líquido dentro da boca? Escapa ou cai pelos lábios?	36	1,00
5 – Você se alimenta de forma mais rápida ou mais lenta do que antes da doença?	31	0,86
6 – Você passou a demorar ou ter dificuldade para comer alimentos sólidos depois da doença?	35	0,97
7 – Depois que você engole ainda ficam restos de alimento na boca?	35	0,97
8 - Você sente que tem muita saliva na boca ou baba com frequência quando está acordado?	33	0,92
9 – Você sente dor para engolir saliva, alimentos ou líquidos?	35	0,97
10 – Sente o alimento/ líquido preso na garganta?	35	0,97
11 – Precisa engolir várias vezes para ajudar o alimento a descer da garganta?	36	1,00
12 – Passou a beber líquidos para ajudar a comida a descer depois da doença?	33	0,92
13 – Sente que sua voz muda durante ou depois da refeição?	31	0,86
14 – Você tosse ou pigarreia durante a refeição?	34	0,94
15 – Se sente sufocado ou com dificuldade para respirar ao se alimentar?	36	1,00
16 – Você engasga frequentemente?	36	1,00
	**IVC – T**	0,96

Sendo assim, pode-se considerar o protocolo validado em sua aparência e conteúdo, já que obteve IVC – T de 0,92.

## DISCUSSÃO

A partir dos artigos selecionados para este estudo, foi possível a criação do protocolo de rastreio multiprofissional, considerando os principais aspectos relacionados à fase oral e faríngea da deglutição.

A validação deste protocolo se deu mediante a análise pelos juízes especialistas em HIV/ Aids assim como pelo público-alvo que irá aplicá-lo. A construção de materiais educativos validados é importante para uniformizar as condutas no cuidado do paciente, sendo indispensável a participação de todos os profissionais^(13)^.

A validação de faces é uma medida que avalia o grau em que os entrevistados consideram a construção e o conteúdo de um teste e seus itens como relevantes para o contexto no qual o instrumento será aplicado. Este meio de validação é considerado fase importante na adaptação de questionários^(28)^ pois permite avaliar a concordância de juízes especialistas e público-alvo sobre cada item individualmente de um questionário e também do instrumento como um todo. É importante ressaltar que este método é o início de um processo que deve englobar outros tipos de validações e confiabilidade^(28)^.

É considerada uma construção robusta do protocolo de pesquisa quando há concordância entre a maioria dos avaliadores. Neste processo, são analisadas as sugestões dos participantes quanto à substituição de termos e reformulação de informações, visando o aperfeiçoamento do material^(16)^. Na análise dos dados foi utilizado o Índice de Validade de Conteúdo.

Os especialistas para julgamento do instrumento criado neste estudo incluíam profissionais da área de Fonoaudiologia, Enfermagem, Nutrição e Medicina, com curso mínimo de mestrado e pelo menos três anos de experiência clínica em cuidados com pacientes com HIV. Não foi encontrada na literatura nenhuma padronização oficial para escolha dos juízes.

Na validação por juízes especialistas, os resultados evidenciaram que 85,7% da amostra foi constituída por indivíduos do gênero feminino. Com relação à faixa etária, influenciada pelos critérios de inclusão do estudo, foi encontrada uma média de 44,1 anos e DP de 11,67.

Foram escolhidos peritos de diferentes áreas da saúde, visto o perfil multiprofissional do protocolo, que atuam diretamente com o paciente em avaliações clínicas e terapia. Todos os convidados aceitaram participar da pesquisa, sendo 3 fonoaudiólogos (42,8%), 2 enfermeiros (28,6%), 1 médico (14,3%) e 1 nutricionista (14,3%). Com relação ao tempo de formação acadêmica, percebe-se que a grande maioria dos juízes possui até 15 anos (42,8%) de formado.

Devido à titulação mínima utilizada como critério de inclusão, observa-se pouca diferença percentual entre participantes com titulação de mestrado (57,1%) e doutorado (42,9%). No que diz respeito à área de trabalho, a grande maioria dos juízes (85,7%) dedica-se tanto à docência quanto à assistência clínica. Em relação à produção bibliográfica, 5 dos avaliadores (71,4%) possuem artigos publicados em periódicos.

Após análise quantitativa das respostas dos juízes na primeira fase, constata-se que dois itens não atingiram o IVC – I mínimo de 0,78. São eles: “6 – Você demora ou tem dificuldade para comer alimentos duros?” (0,71) e “16 – A comida ou bebida vai para o “lugar errado” quando você engole?” (0,71). A revisão desses itens foi feita a partir da análise qualitativa das sugestões dos especialistas.

O item “Você demora ou tem dificuldade para comer alimentos duros?” foi modificado de acordo com a sugestão de uma das especialistas de substituir a nomenclatura utilizada “alimentos duros” por “sólidos”. Além disso, foi aceita a sugestão de outro juiz de utilizar palavras temporais que indiquem que a dificuldade que o indivíduo está apresentando não é prévia à doença, e sim que passou a ocorrer após seu diagnóstico.

Já o item “A comida ou bebida vai para o “lugar errado” quando você engole?”, foi acatada a sugestão de alterar a forma de descrever esta dificuldade, sendo utilizada então “Você engasga frequentemente?”, englobando a frequência que esse evento ocorre, para que assim seja considerado um problema.

Além dessas alterações obrigatórias, também foi realizado um levantamento de todas as sugestões apresentadas, ainda que com IVC – I considerado dentro do padrão adequado, possibilitando melhorias no protocolo criado, mediante a heterogeneidade de experiências dos juízes participantes. Sendo assim, foram alteradas palavras visando tornar as perguntas mais abrangentes e claras; também foram acrescentadas palavras que deixem as perguntas mais autoexplicativas e que representem a temporalidade que a pergunta visa abranger.

Chama a atenção também a análise do item 4: “Há dificuldade de manter a comida/ líquido dentro da boca? Escapa ou cai pelos lábios?”, para o qual os 7 juízes assinalaram a opção “altamente relevante”. Isso pode ser explicado devido ao fato de alterações neurológicas possibilitarem diminuição do tônus muscular, que consequentemente causa uma maior dificuldade no controle oral do bolo alimentar e possibilidade de atraso na sequência faríngea da deglutição, proporcionando uma maior chance de escape prematuro desse bolo tanto anterior como posterior e, consequentemente, risco de broncoaspiração^(7)^. Além disso, essa dificuldade é muito encontrada em avaliações objetivas de paciente com alterações neurológicas^(29)^.

Após as alterações realizadas, o protocolo foi novamente enviado aos juízes especialistas para o seguimento da validação de aparência e conteúdo.

Na análise quantitativa da segunda fase de validação pelos juízes, com o protocolo modificado, observou-se IVC – I acima de 0,78 para todos os itens, bem como IVC – T de 0,92. Sendo assim, foi possível iniciar a terceira fase da validação, com a avaliação do público-alvo.

É importante observar também que a pergunta “Precisa engolir várias vezes para ajudar o alimento a descer da garganta?”, durante essa segunda avaliação, recebeu as 7 avaliações “altamente relevante”. Essa marcação pode ser justificada devido à alteração na forma como a pergunta foi reescrita, possibilitando maior acessibilidade do vocabulário à realidade da população do estudo. Além disso, pode ser encontrado na literatura especializada que a sensação de bolo na garganta tem grande relação com a dificuldade de deglutição vista em exames objetivos, levando ao maior risco para disfagia orofaríngea^(29)^.

A fase seguinte da validação é de extrema importância, já que o público-alvo da pesquisa foi composto por indivíduos que normalmente têm o primeiro contato com o paciente e que, portanto, serão responsáveis por aplicar o instrumento. Essa população deve ser capaz de compreender as perguntas que o questionário contempla, bem como de reproduzi-las para a população do estudo. Além disso, é o momento de analisar opiniões e sugestões para a elaboração deste material. Esse é o momento de verificar como o material foi entendido por essas pessoas e identificar o que não ficou claro^(13)^.

Com relação a esse público-alvo, os resultados mostraram que 86,1% da amostra foi constituída por indivíduos do gênero feminino. Com relação à faixa etária, foi identificada uma média de 30,1 anos e DP de 7,17.

Contou-se com a avaliação de 10 universitários dos últimos períodos de diferentes cursos das ciências da saúde (27,8%), 15 profissionais com formação técnica (41,7%), além de 7 médicos (19,4%) e 4 enfermeiros (11,1%).

Feita a análise quantitativa dessa população, observa-se que nas perguntas “5 - Você se alimenta de forma mais rápida ou mais lenta que antes da doença?”, “6 – Você passou a demorar ou ter dificuldade para comer alimentos sólidos depois da doença?”, “7 - Depois que você engole ainda ficam restos de alimento na boca?”, “8 - Você sente que tem muita saliva na boca ou baba com frequência quando está acordado?”, “9 - Você sente dor para engolir saliva, alimentos ou líquidos?”, “10 - Sente o alimento/ líquido preso na garganta?”, “12 - Passou a beber líquidos para ajudar a comida a descer depois da doença?”, “13 - Sente que sua voz muda durante ou depois da refeição?” e “14 - Você tosse ou pigarreia durante a refeição?” pelo menos 1 participante considerou pouco relevante cada uma dessas questões, porém não houve necessidade de alteração, já que a maioria dos avaliadores classificou os itens como bastante ou altamente relevantes.

Além disso, o tempo de trânsito oral lento, dificuldade na manipulação do alimento, resíduos em cavidade oral, estase de saliva, odinofagia, sensação de alimento parado, voz molhada e tosse^(29)^, a que se referem as questões, são sintomas frequentemente encontrados em indivíduos com dificuldade de alimentação.

Após a etapa de validação pelo público-alvo, observou-se através da análise quantitativa que não houve necessidade de modificações do questionário, devido aos valores de IVC – I acima de 0,78 para todos os itens e IVC – T de 0,92. Sendo assim, pode-se considerar o protocolo validado em sua aparência e conteúdo, atendendo sua finalidade.

Vale destacar que ainda são necessárias outras etapas de validação para que seja possível utilizar o instrumento na prática clínica.

## CONCLUSÃO

O protocolo de rastreio multiprofissional de disfagia em pacientes com infecção HIV é um instrumento de triagem, que visa a identificação precoce do risco para dificuldade de deglutição e interdição de uma possível complicação pulmonar.

Este protocolo pode ser considerado validado do ponto de vista de aparência e conteúdo, uma vez que apresentou um IVC total de 0,92 na validação dos juízes especialistas e IVC total de 0,96 a partir da validação pelo público-alvo, constatando-se, assim, um IVC do protocolo de 0,94. Sendo assim, podemos considerar o instrumento resolutivo, com capacidade de cumprir o que se propõe.

Diante das sugestões e contribuições de todos os participantes durante o processo de validação, o protocolo passou por modificações de linguagem e estruturação, tornando-o mais abrangente e qualificado para utilização no dia-a-dia das pessoas que atuam com esses pacientes abordados na pesquisa.

É importante ressaltar que o protocolo foi validado em seu conteúdo e aparência. Outros estudos são necessários para verificar sua utilização, incluindo aplicação experimental em larga escala com abrangência de diferentes profissionais e instituições (validação interna e externa e confiabilidade) para que o instrumento possa ser utilizado na prática clínica.

## Material Suplementar

Este artigo acompanha material suplementar.

Apêndice 1 Protocolo de Rastreio de Disfagia em Pacientes HIV

Apêndice 2 Formulário para Validação da Aparência e Conteúdo

Este material está disponível como parte da versão online do artigo na página: https://www.scielo.br/j/codas
